# The Societal Cost of Behaviors of Concern Among Individuals with Intellectual and Developmental Disabilities Residing in Small Residential Group Homes

**DOI:** 10.3390/ijerph22020168

**Published:** 2025-01-26

**Authors:** Tricia J. Johnson, Hugh Vondracek, Teresa Moro, Cameron D. White, Sarah H. Ailey

**Affiliations:** 1 Department of Health Systems Management, Rush University, Chicago, IL 60612, USA; 2 Department of Community, Systems and Mental Health Nursing, Rush University, Chicago, IL 60612, USA; hugh.vondracek@gmail.com (H.V.); sarah_h_ailey@rush.edu (S.H.A.); 3 Office of Nursing Research and Scholarship, College of Nursing, Rush University, Chicago, IL 60612, USA; 4 Department of Social Work, Rush University, Chicago, IL 60612, USA; teresa_moro@rush.edu; 5 College of Nursing, Rush University, Chicago, IL 60612, USA; white.camerond@gmail.com

**Keywords:** intellectual disability, developmental disability, cost and cost analysis, group homes, small residential group homes, societal costs, behaviors of concern

## Abstract

Engagement in behaviors of concern (BoCs) by adults with intellectual and developmental disabilities (IDDs) living in small residential group homes can negatively impact the health, safety, and quality of life of the individuals themselves and others living and working in the home. Little is known about the societal cost of BoCs. The objective of this study was to quantify the cost of BoCs for residents, residential group homes, and public and healthcare services in terms of different behaviors. This descriptive study used incident and monthly behavior-tracking reports collected from small residential group homes for six months prior to implementing a social problem-solving intervention aimed at decreasing BoCs. The mean cost per BoC incident per resident was USD 80 ± 601. Health and safety incidents had the highest cost, followed by begging. BoCs impose costs on small residential group homes, public and healthcare services, and residents themselves.

## 1. Introduction

Nearly 4.3 million (1.8%) individuals age 18 and older in the United States have an intellectual or developmental disability (IDD), and 13.9% reside in small residential group homes managed by agencies serving adults with IDDs [1,2]. Behaviors of concern (BoCs), including physical and verbal aggression, destructive and socially offensive behaviors, and other BoCs, are common in group homes [3,4,5]. Residential staff have reported that they may observe BoCs several times per week or more [6].

BoCs are not only an issue for the residential group homes themselves but also a public health issue. Some behaviors lead to outside intervention from law enforcement, emergency medical technicians, and other public, social and healthcare services. BoCs are a leading reason for psychiatric admissions for individuals with IDDs [7]. Furthermore, 19.5% of individuals in the federal prison system and 30.9% of individuals in jail have a cognitive disability, rates that are four to six times higher than the prevalence of a cognitive disability in the general population [8].

As individuals with IDDs age, they often lose their familial support due to parental illness or death. Over the past 35 years, there has been a tremendous shift from large residential facilities to small-staffed residential group homes (i.e., homes with eight or fewer residents and oversight by residential staff), with the number of individuals with IDDs residing in group homes with six or fewer people increasing twentyfold, from 20,400 in 1977 to 427,947 in 2017 [8]. More than 220,000 small group homes were in operation in 2017, and 83% of individuals with IDDs who live in nonfamily residential facilities now reside in small group homes [9]. The small group home environment can decrease the social distance between residential staff and residents and encourage social networks among adults with IDDs [10].

Once in a small residential group home, adults with IDDs receive support from direct support professionals (DSPs), who provide day-to-day care and work with them on their personal needs and goals as well as attending to health and safety concerns. DSPs’ training, certification, and education requirements vary by state, with some states requiring an eighth grade education and others requiring a high-school diploma or GED. Typical DSP training includes skills in participant empowerment, relationship building, conflict resolution, crisis intervention, facilitation of services, vocational support, and person-centered goals and objectives to help individuals with IDDs be part of their families, communities, and friends; training is increasingly competency-based [11]. In 2006, the Department of Health and Human Services projected a need for more than one million new DSP positions by 2022. This shortage is now considered a crisis, exacerbated by high staff turnover and vacancies within the DSP workforce [12]. Discussions regarding turnover note the interaction between the complexity of caring for adults with IDDs, including those with complex behavioral health needs, the lack of standardized training and requirements, and issues including the low wages for the DSP workforce [13].

BoCs incur costs for the residential group home, the residents, the DSPs and other staff, and society, although scant research exists on the costs of these behaviors. State Medicaid programs cover home- and community-based services (HCBS) for individuals with IDDs, including the cost of residential group homes [14]. In 2018, individuals with IDDs accounted for 43% of enrollment in Medicaid HCBS Section 1115 waiver programs and 68% of total expenditures, with an average of USD 46,300 per person annually [15].

When a BoC occurs, agency staff in small residential group homes are expected to address and document the behavior and resolution, diverting time from other valuable activities, which represents an opportunity cost for the staff member and home. For any behaviors, multiple staff members may be involved, including a diverse group who write the behavior plan. These include Board-Certified Behavior Analysts, certified independent practitioners who provide behavior analytic services and intervention plans; qualified intellectual disability professionals (QIDPs), who are responsible for developing and monitoring individualized habilitation plans and ensuring the rights of individuals with IDD are protected; social workers, who are involved in case management and behavior plans; or other staff with expertise in behaviors. For any single incident, these staff may be involved, as well as house managers, who oversee the day-to-day operations and quality of services of the home, DSPs, and agency administration. Guardians or family members may also be involved. Additionally, these behaviors may lead to the involvement of outside entities, including police and social service providers, emergency medical technicians, and healthcare providers, thereby incurring a cost to taxpayers and society.

The issue of the costs of BoCs among persons with IDDs in community residential settings has been addressed since the late 1990s, with notations that persons with IDDs and severe BoCs were then often still housed in institutional settings or together in smaller community residential settings [16]. In later work on costs in small community residential settings, scores on the Aberrant Behavior Checklist, a measure of BoCs, were associated with 26% of the variance in staff costs [16].

Most work addressing costs to date has used the Client Service Receipt Inventory [17]. It has been used in more than 500 studies [18], including many involving persons with IDDs. This inventory is usually completed by social services staff or family members and is a recall of services used over a 12- to 13-week period. Previous studies indicated high agreement with relevant records of service use [19]. The Inventory typically covers background client information, accommodations and living information, employment, and informal carers. It is amended for each study to be relevant to the study setting and population. For persons with IDDs, the Inventory covers issues including living arrangements, use of day services such as vocational and living skills training, medications to manage behaviors, cost of multidisciplinary meetings, use of professional services, hospital care, respite care, and staffing in residential settings. Studies have generally addressed the total costs of health and social care, while the service staff time directly required to manage behaviors has not been well documented [20,21].

Three Cochrane reviews of interventions for outwardly directed BoCs for persons with IDDs have been conducted, and all three reviews found a lack of methodologically sound clinical trials and recommended randomized controlled trials with sufficient power to evaluate reductions in BoCs and cost-effectiveness [22,23,24]. To address these gaps, the Steps to Effective Problem Solving (STEPS) (R01HD086211) was a randomized clinical trial comparing group homes receiving a social problem-solving intervention designed to decrease BoCs in comparison to attention control group homes receiving a nutrition intervention. Evidence suggests the following. (1) Individuals with IDDs are susceptible to BoCs due to deficits in social problem-solving, meaning the ways in which people view problems and their ability to solve them [22,23,24,25,26]. (2) Social problem-solving programs can help people with IDDs to improve their social problem-solving skills and the outcomes are generally better for individuals who have staff members who reinforce the information [22,23,24,25,26,27,28]. (3) Factors in the environment (chaotic, sensory issues, structure/lack of structure) are known to affect behaviors, and group environmental factors are also known to affect interventions. The STEPS addressed each of these issues, including a measure of the group environment for the intervention [29,30].

One component of the STEPS program was to evaluate the cost savings of the STEPS intervention due to a reduced frequency and severity of BoCs for community agencies and for society. Using data collected through the STEPS, the objectives of this paper are to describe a novel approach for quantifying the cost of BoCs and to demonstrate the importance of quantifying the costs in three areas, including costs that are borne by the small residential group homes, including staff time and physical damage (i.e., in-home costs); costs borne by residents themselves (i.e., resident opportunity costs); and costs that are external to the small residential group home, such as costs incurred by police, ambulance, and healthcare providers. In this paper, we address the direct costs related to time spent managing behaviors rather than the overall staff costs, in addition to measuring the costs of health and social service use, as has been performed in prior studies. Additionally, this study adds to the literature by addressing the costs to the persons with IDDs themselves.

## 2. Materials and Methods

This was a descriptive study of the costs of BoCs that occurred between January 2017 and December 2019 in small residential group homes that participated in the STEPS clinical trial. This study was approved by the Rush University Medical Center Institutional Review Board (15020202-IRB01).

### 2.1. Setting

The STEPS intervention was fully manualized and delivered to all eligible and willing residents and staff in participating group homes [30]. Detailed inclusion and exclusion criteria have been previously reported and are briefly described here [30]. Group homes were eligible to participate if they met the following criteria: four or more residents in the home; three or more residents with mild to moderate IDDs agreed to participate; 1+ residential staff agreed to participate; 10+ BoCs documented in incident or monthly behavior reports within the group home in the prior 6 months; and 30% or more of residents had documented BoC incidents or monthly behavior reports in the prior 6 months. Residents were eligible to participate if they met the following criteria: age 18 or older, spoke English, were able to verbally communicate, had mild to moderate IDDs (IQ of 50–75), and had mild to moderate limitations in adaptive functioning as per agency records.

### 2.2. Sample and Procedure

A component of the STEPS was collecting the documentation of BoCs, including incident reports and monthly behavior-tracking reports. These data were collected on enrolled study participants with IDDs for six months before and six months after participation in the intervention, and this study used data collected for the six months prior to participation [30]. Incident reports with insufficient information to classify the incident into a behavior type were excluded from the analysis (*n* = 61).

### 2.3. Behavior of Concern Incident Reports

The incident reports documented BoCs that were either unexpected behaviors or were severe enough to require formal documentation, as determined by the DSPs and house managers. Only incident reports for individuals participating in this study were collected. Names and identifying locations were redacted from the incident reports, and the reports were labeled with the unique participant identification number. A standardized data entry form was created in REDCap with a common coding scheme to collect information on the incident duration, individuals involved, and actions taken to address the BoCs, utilizing incident reports from different group homes, and the pre-defined data elements required for the cost analysis [31,32]. Each incident was classified into a behavior type based on a predefined list of incidents in the standardized data entry form using either information in free text or the incident types on the agency’s incident report forms. Incident reports that mentioned more than one type of BoC were classified as having a combination of behaviors. The study team reviewed the incident reports and behavior reports from multiple agencies to determine a list of key terms and classifications. Research assistants were trained in the data entry procedures, and they extracted data from the reports and entered data into the REDCap study database.

### 2.4. Monthly Behavior-Tracking Reports

The monthly behavior-tracking reports recorded the occurrence and nature of the expected BoCs (i.e., behaviors that are included in a behavior plan). Each resident of a small group home with one or more consistent problem behaviors had a behavior plan developed by a Board-Certified Behavior Analyst, QIDP, social worker, or other qualified staff. Behavior-tracking forms were completed by DSPs, who documented how many incidents of each behavior occurred for a resident each day. Monthly behavior-tracking reports were generated from the forms. Unlike the BoCs documented in incident reports, the monthly behavior-tracking reports did not document the time spent to resolve or manage the documented behaviors.

### 2.5. Measures

#### 2.5.1. Types of Behaviors of Concern

Behavior typological groups were created based on the BoC type categories included in the incident reports from two agencies and further refined through a review of the free-text descriptions of the BoCs reported in the incident reports from a third agency. Two research assistants reviewed the classification and conducted data entry from the first three agencies for consistency. The behavior types are described in Table 1.

The BoCs reported through the behavior-tracking reports were entered into a database using the behavior classifications from the reports and other information included in the report, such as the location (home, day program, work setting, transportation between settings) and shift. The cost of each behavior was based on the median resolution time from a survey of DSPs and QIDPs (described below). If there was a difference between the estimations from the DSPs and the QIDPs, we used the median duration reported by the DSPs, since they are responsible for resolving these behaviors on a day-to-day basis.

Development of behavior support plans and training of support staff in the plans are more complicated and time-consuming depending on the types and numbers of behaviors being addressed. We have provided a descriptive table of the behavior plans. We also recognize that the staff time taken to document behaviors (even zero behaviors) increases with each additional behavior to track. We assumed the time to develop behavior support plans and subsequently report the behaviors, collect data, and develop monthly reports generated a fixed base cost for the individuals with IDDs with a behavior support plan and excluded this fixed cost from the analysis.

#### 2.5.2. Data Validation

To validate the information reported in the incident reports, including the approximate amounts of time required to manage the behaviors reported in the incident reports, and to determine the approximate amount of time taken to manage the behaviors reported in the monthly behavior-tracking reports, two surveys were sent electronically to DSPs (*n* = 10) and QIDPs (*n* = 9) via the agency’s human resources representative or director. DSPs were surveyed because they complete the behavior-tracking forms and manage the behaviors in the home. QIDPs were additionally surveyed regarding their estimation of these times. One survey collected information related to the incident reports, and the second survey collected information related to the behavior-tracking reports. The incident report survey collected information about the typical amount of time needed to resolve different types of incidents documented in the incident reports, including the amount of time required to resolve each behavior type (i.e., threshold minimum duration) and the amount of time required to complete the incident report. The behavior-tracking report survey collected information about the typical amount of time required to resolve behaviors documented in the monthly behavior tracking reports, including specific questions about the amount of time taken to resolve each type of behavior.

The minimum duration for reported incidents was estimated as the median typical duration for the corresponding behavior type that was collected through the incident report survey as a conservative estimate of the incident duration. If there was a difference between the estimations from the DSPs and the QIDPs, we used the lower of the two medians. For incident reports that had missing start and/or end times, or incidents with durations reported less than the threshold minimum duration, the threshold minimum duration was used. For incident reports with missing staff member roles, the role was classified as unknown.

#### 2.5.3. Cost Domains

Resources and their costs were classified into three domains: in-home, resident opportunity, and public and healthcare services. The in-home cost domain included the time spent by residential staff and other resources involved in the BoC, the time taken by staff to resolve the incident, property damage as reported by the residential staff, and time to document via the incident report. Although residential staff costs may be fixed regardless of BoCs, the time spent addressing and documenting these BoCs takes away time from adding value to residents’ lives through other activities. Thus, reducing BoCs creates value by redirecting staff time to resident care, even without decreasing labor costs. Staff members were classified as DSP, house manager, QIDP, program coordinator, Board Certified Behavior Analyst, registered nurse, agency director, and unknown staff role. The resident opportunity cost domain included the time of all the residents with IDDs who were involved in the BoC. The public and healthcare services cost domain involved in the BoC included the costs associated with the following services: public services, including a 911 call, police response, fire department response, ambulance response, ambulance transportation, police arrest; disability services, including contact with pre-admission screening agencies that provide 24-hour help to individuals with IDDs, families and providers, family guardian contact, state guardian contact; and healthcare services, including emergency department visit, hospitalization, a call with a physician, a physician visit, and continued physician monitoring.

#### 2.5.4. Quantification of Costs

For each of the three domains, the costs were assigned to each resource involved in or responding to the BoC (Table 2). To determine the costs in the in-home cost domain, the duration of the incident (hours) was multiplied by the respective hourly wage rate for each in-home staff member involved in the BoC. The 2019 national median hourly wage rate from the US Bureau of Labor Statistics was used for each role that corresponded to an occupation in the Standard Occupational Classification System (SOCS) [33]. The SOCS does not have an occupational category for DSPs, so we used the US national minimum wage in 2019 as the hourly wage for this role. The hourly wage rate for unknown roles was based on the average hourly wage rate for the DSP and house manager. The in-home property damage costs were calculated based on the narrative description of the cost of damages. The cost of each in-home staff member involved in the incident, property damage costs and documentation costs were summed to calculate the total in-home costs.

To determine the costs in the resident opportunity cost domain, the resident opportunity cost (i.e., the value of the time of the residents involved in the BoC) was calculated by multiplying the duration of the incident (hours) by the US minimum hourly wage rate by the number of residents involved.

To determine the costs in the public and health services cost domain, the cost of each public and healthcare service involved in the incident was summed to calculate the total public and healthcare services cost. The in-home, client opportunity, and public and healthcare service costs were summed to calculate the societal cost per incident. The total 6-month societal cost per resident (for the aggressor with the BoC) was calculated by summing the societal costs for each incident. All the costs were based on the best available literature for the cost of each service and were valued in 2019 US dollars. Because the behaviors reported in the monthly behavior reports are less severe, the costs were limited to the time spent by the DSP and resident on resolving the behavior.

### 2.6. Analysis

For each cost domain and the overall societal cost, the per-incident mean cost and standard deviation were computed for the BoCs reported in incident reports and monthly behavior reports. The mean cost (and standard deviation) of BoCs per resident per month by behavior type was calculated for the BoCs reported in the monthly behavior reports. SPSS v.23 command syntax was used for all the statistical analyses (Armonk, NY).

## 3. Results

**Figure 1:**
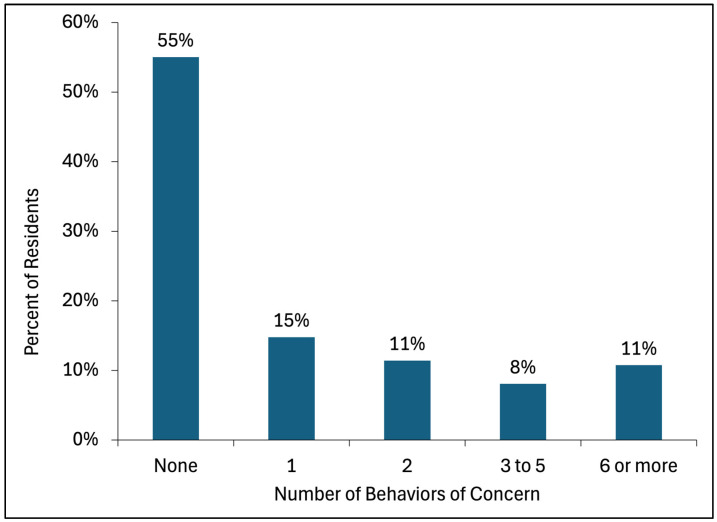
Distribution of residents by number of behaviors of concern in incident reports.

The sample included 35 small group residences and 149 residents enrolled in this study, with a total of 508 incident reports from 67 residents and 595 participant months of behavior-tracking reports from 119 residents. Overall, 67 (45%) had at least one BoC documented in an incident report, and 11% had six or more BoC incidents during a 6-month period of time (Figure 1). For residents with at least one BoC, the median number of BoC incidents was two (interquartile range (IQR): one, five). Additionally, 119 (69%) residents had a behavior plan. The median number of BoCs in a behavior plan was 5 (IQR: 0, 21).

Of the 508 BoCs, verbal aggression was the most common (34%), followed by “other” behaviors (31%), combination behaviors (11%), health and safety behaviors (9%), and physical aggression (6%). Overall, the mean societal cost per incident was USD 80 ± 601 (Table 3). This was primarily due to costs to public and healthcare services (USD 38 ± 593), resident opportunity costs (USD 21 ± 52), and in-home incident costs (USD 19 ± 36). The most expensive single BoC within the typology was health-and-safety-related incidents, with an associated societal cost of USD 369 ± 1899, of which USD 323 ± 1894 was borne by public and healthcare services, USD 24 ± 40 was in-home incident costs, and USD 20 ± 41 was resident opportunity costs. The second highest cost incident was begging, with a mean societal cost of USD 186 ± 108. Begging and elopement had the highest in-home incident costs of USD 80 ± 47 and USD 40 ± 45, respectively. Table S1 (Supplementary Materials) reports the median cost by BoC in the incident reports.

The most common behavior types tracked in the monthly behavior plans included verbal aggression (69% of residents with behavior plans), physical aggression (46%), and property damage (24%) (Table 4). Other behavior types were common and were found for 77% of residents with behavior plans. While rare, elopement required the longest time to resolve and had the highest cost (USD 6.09 per behavior) from a societal perspective, followed by verbal aggression, property damage and physical aggression behaviors, each with a per-behavior cost of USD 3.63 to resolve.

Table 5 reports the mean cost per month for the BoCs documented in the behavior plans. Overall, the behaviors documented in the behavior plans were observed in 53% of months (823/1560). The mean cost per behavior per month, including months where the behavior was not observed, was USD 12 ± 48. The cost per participant behavior per month ranged from USD 4 ± 8 for property damage to USD 23 ± 85 for other behaviors. The cost per behavior per month increased to USD 23 ± 64 when limited to months when the behavior was observed, with the cost per participant behavior per month ranging from USD 10 ± 9 for health and safety behaviors to USD 39 ± 108 for other behaviors. Table S2 reports the median monthly cost per BoC in the behavior plans.

## 4. Discussion

BoCs are an important public health issue that is costly for the residents, co-residents, staff, and society overall. The main objective of this paper was to describe a methodology for characterizing the types and the associated costs of BoCs by individuals with IDDs living in small residential group homes. Most adults with IDDs who do not live with families reside in small residential group homes; however, scant data exist on the cost of the BoCs that occur in these homes. The costs of these behaviors must be considered in the design of interventions and programs to reduce the occurrence and severity of the behaviors. We found that the societal cost of a BoC is USD 80 on average, although there is substantial variation in the costs, depending on the behavior type. However, these costs likely represent a lower bound of the costs borne by the small residential group homes, residents, and public and healthcare service providers. For example, there is limited literature on valuing the opportunity cost of individuals with IDDs, and therefore, we used the minimum wage rate as a lower bound of the value of their time. Additionally, the average hourly wage for DSPs may have been higher than the minimum wage, thereby representing a lower bound on the hourly wage rate for these staff members. Finally, any costs that were incurred after documenting a BoC incident or behavior plan were not captured in the analysis.

Recognizing that we worked with homes with high rates of behaviors, we found that 45% of residents had BoC incidents over 6 months, and of those, nearly one-quarter (24%) had six or more incidents, similar to prior findings from studies on aggressive BoCs [3,4,5]. This concentration in costs suggests that designing interventions to reduce these behaviors for individuals with multiple incidents may be a cost-effective strategy. While many of these behaviors impose a cost on the residential home via staff time taken to resolve and document the BoC, many behaviors also impose an additional cost on public and healthcare services, and these costs should not be overlooked.

In addition to providing residential services for persons with IDDs, public services, including 911 calls and police and ambulance responses, impose additional costs to taxpayers and society overall, and public services are funded through different streams than residential services. Reducing BoCs that require these services could have a significant impact on both the residential homes and the public services. In a study of police involvement to address BoC crises in adults with intellectual disabilities, Raina and colleagues found that police were most frequently called to resolve issues related to physical aggression, followed by suicidal behavior and verbal aggression, with 11% of police calls resulting in an arrest, 55% resulting in an emergency department visit and only 34% resolved on-site [34]. Additionally, having a history of legal involvement is a predictor of subsequent incidents requiring police involvement [35]. Our findings demonstrate that incidents requiring the police and other public and healthcare services are the costliest, and interventions that reduce their frequency or intensity could also result in societal cost savings.

Although the greatest attention is placed on BoCs requiring the completion of an incident report, behaviors that are expected and less severe also have a cost to the home and resident. Interventions to reduce the frequency and severity of these behaviors could ultimately increase the time residential staff spend on other individualized support of residents and ensure that residents reach and maintain their personal goals and potential. Interventions that improve skills in social problem-solving and decrease BoCs may also have the spillover effect of reducing residential staff turnover, which was 43% in 2019, although more research is needed [36,37].

Providing training to persons with IDDs who are able and living in small group homes on skills to address their own issues that may lead to BoCs is important for their well-being and achieving their goals. Providing training in problem-solving skills fits with the paradigm of positive behavior support. Ensuring that residential home staff are adequately trained for managing and preventing BoCs, particularly those behaviors that either occur most frequently or are most costly, is important for the well-being of residents and staff. Providing specific training to DSPs fits with efforts to provide competency-based training to this workforce, particularly training in the competencies for providing person-centered support [38].

### Limitations

There are several limitations that should be considered. First, the records of the occurrence of BoCs were retrospective and relied on an incident report being created or documentation in a monthly behavior report. The types and severity of the behaviors that were documented in the incident reports versus the monthly behavior reports may have varied among residential homes and among staff members within and across homes, and this variation may have introduced unobserved heterogeneity in the documented BoCs and their respective costs. Additionally, detailed data on the duration of involvement of police, paramedics, and other public and healthcare service providers were not collected, and therefore, we estimated the cost of these services as fixed costs rather than variable costs. We did not have detailed information about the actual costs that resulted from a BoC, such as home repairs or replacement of belongings, that were incurred after the incident report was written. We also did not have information about cases where a staff member from another residential home came to “help out” at the residential home during or after an incident that required substantial staff time to resolve. We specifically recruited residential homes with high rates of BoCs, and as such, the frequency of these behaviors may not be representative of all small residential group homes. Reducing BoCs may have improved staff retention; however, we did not have access to data on turnover and staff absences. Finally, reducing BoCs may have improved the psychological well-being of other residents in the homes. Future studies should examine whether interventions to reduce BoCs have downstream effects on staff satisfaction and retention as well as resident quality of life and well-being.

Future work should evaluate the protocols and decision-making processes used for assessing a BoC and determining whether it requires documentation via an incident report or a behavior-tracking form. Each residential home used a different format for its incident report, introducing potential variation in the granularity of the information describing incidents. Although a standardized data collection protocol was utilized to abstract information from the incident reports, there may be unobserved differences in the information collected by homes. We classified BoCs that included multiple types of behaviors as “combination” behaviors due to the small numbers of incidents with the same combination of behavior types. While these BoCs represented a relatively small proportion of all the incidents, they incurred a relatively high cost on average, but with substantial variation. Future work should examine the prevalence and cost of specific combinations of BoCs that co-occur in a larger sample. Finally, the information documented in the monthly behavior reports was limited to the occurrence and type of behavior. To address this limitation, we surveyed residential home staff about the typical time required to resolve behaviors, and therefore, were not able to capture the variation in the resolution time within a particular behavior type.

## 5. Conclusions

The societal cost of BoCs for individuals with IDDs living in small residential homes is substantial, incurring costs to the residential home, public and healthcare services, taxpayers and residents themselves. Both the cost per behavior and the frequency of behaviors contribute to the overall cost to society. Interventions designed to reduce the occurrence and/or severity of BoCs have a multitude of benefits for all stakeholders.

## Figures and Tables

**Table 1:** Types of behaviors of concern in the cost analysis.

Behavior Type	Description
Begging	Asking for something in a disruptive way
Elopement	Leaving or wandering from the home, day program, or other site
Fire alarm pull	Pulling a fire alarm when there is no fire
Health and safety	Any incident that endangers the individual’s own health or safety, such as pica, hoarding and then eating food, and other self-injurious behavior
Inappropriate sexual behavior	Actions including sexually suggestive comments, touching/groping or any type of sexual assault
Lying	Not telling the truth
Physical aggression	Physical actions, including hitting, pushing and kicking
Property damage	Any behavior that results in property damage, such as a hole in the wall, broken chair or broken television
Theft of property	Taking any item that does not belong to the resident, whether it is from another resident or business
Verbal aggression	A verbal action, including yelling, cursing or name calling
Other behaviors	Any behavior that does not fit in another category
Combination behaviors	Any behavior that includes a combination of behavior types

**Table 2:** Resource costs.

Resource	Unit	Cost per Unit
In-home incident and documentation costs		
Direct support professional	Per hour	USD 7.25
Unknown staff role	Per hour	USD 9.13
House manager	Per hour	USD 11.00
Qualified intellectual disability professional (QIDP)	Per hour	USD 16.85
Program coordinator	Per hour	USD 21.00
Behavior analyst	Per hour	USD 22.23
Registered nurse	Per hour	USD 35.24
Agency director	Per hour	USD 49.63
Property damage	As reported	Per incident
Resident opportunity costs	Per hour	USD 7.25
External costs		
911 call	Per episode	USD 6
Police response	Per episode	USD 125
Fire department response	Per episode	USD 250
Ambulance response	Per episode	USD 250
Ambulance transportation	Per episode	USD 2500
Arrest	Per episode	USD 2000
PAS contact	Per hour	USD 21.03
Family guardian contact	Per hour	USD 7.25
State guardian contact	Per hour	USD 31.25
Emergency department visit alone	Per visit	USD 633
Hospitalization	Per stay	USD 12,594
Physician call	Per episode	USD 23.46
Physician visit	Per visit	USD 76.15
Continued monitoring by physician	Per day	USD 23.46

**Table 3:** Mean cost (SD) per behavior of concern in the incident reports by type of behavior and cost domain.

Behavior Type	*n*	In-Home		Resident Opportunity Cost (USD)	Public and Healthcare Services Cost (USD) M (SD)	Societal Cost (USD) M (SD)
		Incident Cost (USD) M (SD)	Documentation Cost (USD) M (SD)			
Begging	3	80 (47)	1 (0)	105 (62)	0 (0)	186 (108)
Elopement	9	40 (45)	6 (0)	28 (63)	18 (33)	91 (106)
Health and safety	48	24 (40)	2 (0)	20 (41)	323 (1894)	369 (1899)
Inappropriate sexual behavior	5	12 (6)	4 (0)	6 (2)	0 (0)	23 (7)
Lying	6	11 (16)	2 (0)	11 (21)	0 (0)	24 (37)
Physical aggression	30	16 (7)	3 (0)	8 (8)	8 (46)	36 (48)
Property damage	7	13 (6)	3 (0)	4 (2)	0 (0)	20 (5)
Theft of property	13	9 (5)	2 (0)	3 (2)	10 (35)	24 (39)
Verbal aggression	171	21 (38)	2 (0)	32 (68)	0 (0)	55 (105)
Combination	57	28 (37)	2 (0)	32 (50)	39 (282)	101 (299)
Other	159	11 (34)	2 (0)	10 (39)	8 (73)	31 (102)
TOTAL	508	19 (36)	2 (1)	21 (52)	38 (593)	80 (601)

Costs are reported as the mean (SD) per BoC.

**Table 4:** Mean cost per behavior of concern in the behavior plans by type of behavior.

Behavior Type	Number with Behavior Tracked in Behavior Plan *n*N (%)	Time to Resolve (Min)	In-Home Cost (USD)	Resident Opportunity Cost (USD)	Societal Cost (USD)
Elopement	14 (12)	25	3.05	3.05	6.09
Health and safety	14 (12)	15	1.81	1.81	3.63
Hoarding	4 (3)	2	0.58	0.58	1.16
Lying	3 (3)	2	0.58	0.58	1.16
Inappropriate sexual behavior	9 (8)	15	1.81	1.81	3.63
Physical aggression	55 (46)	10	1.23	1.23	2.47
Property damage	28 (24)	15	1.81	1.81	3.63
Self-injurious	20 (17)	15	1.81	1.81	3.63
Theft of property	16 (13)	15	1.81	1.81	3.63
Verbal aggression	82 (69)	15	1.81	1.81	3.63
Other	92 (77)	2	0.58	0.58	1.16

**Table 5:** Mean cost per month for behaviors of concern in the behavior plans by type of behavior.

Behavior Type	Per Month, Including Months Where Behavior Was Not Observed			Per Month, Only Months Where Behavior Was Observed		
	Participant Months *n*	Number per Resident per Month M (SD)	Societal Cost (USD) M (SD)	Participant Months	Number per Resident per Month M (SD)	Societal Cost (USD) M (SD)
Elopement	66	1.3 (2.6)	8 (16)	22	3.9 (3.3)	24 (20)
Health and safety	46	1.4 (2.2)	5 (8)	24	2.7 (2.4)	10 (9)
Hoarding	24	0.2 (0.5)	0 (0.6)	4	1.3 (0.5)	1 (1)
Inappropriate sexual behavior	38	3.0 (6.8)	11 (25)	13	8.7 (9.4)	32 (34)
Lying	17	14.5 (17.3)	17 (20)	12	20.5 (17.3)	24 (20)
Physical aggression	255	2.1 (4.4)	5 (11)	114	4.8 (5.6)	12 (14)
Property damage	126	1.5 (3.3)	4 (8)	45	4.3 (4.2)	11 (10)
Self-injurious	98	3.3 (7.3)	12 (27)	48	6.8 (9.3)	25 (34)
Theft of property	63	2.1 (3.5)	8 (13)	36	3.7 (4.0)	13 (15)
Verbal aggression	387	9.2 (17.8)	11 (21)	247	14.4 (20.6)	17 (24)
Other	440	19.7 (72.9)	23 (85)	258	33.6 (92.8)	39 (108)
Overall	1560	8.9 (40.5)	12 (48)	823	16.9 (54.6)	23 (64)

## Data Availability

The data presented in this study are available on request from the corresponding author due to ongoing data analysis.

## Supplementary Materials

The following supporting information can be downloaded at https://www.mdpi.com/article/10.3390/ijerph22020168/s1, Table S1. Median cost per behavior of concern in the incident reports by type of behavior and cost domain. Table S2. Median cost per behavior of concern in the behavior plans by type of behavior and cost domain.
