# Supplementary material for: The Societal Cost of Behaviors of Concern Among Individuals with Intellectual and Developmental Disabilities Residing in Small Residential Group Homes
Source: Int J Environ Res Public Health. 2025 Jan 26;22(2):168. doi: 10.3390/ijerph22020168 (PMC11855932; doi:10.3390/ijerph22020168)
Supplement: Supplementary file 1 [file ijerph-22-00168-s001.zip › ijerph-3394864-supplementary.pdf]

Supplementary Materials

Table S1. Median Cost per Behavior of Concern on Incident Report by Type of Behavior and Cost Domain

Behavior Type	n	In-Home		Resident Opportunity Cost ($) Med (IQR)	Public and Healthcare Services Cost ($) Med (IQR)	Societal Cost ($) Med (IQR)
		Incident Cost ($) Med (IQR)	Documentation Cost ($) Med (IQR)			
Elopement	9	29 (13, 33)	6 (6, 6)	6 (3, 9)	0 (0, 0)	59 (27, 114)
Health and safety	48	6 (3, 25)	2 (2, 2)	3 (1, 13)	0 (0, 0)	11 (6, 62)
Inappropriate sexual behavior	5	10 (10, 12)	4 (4, 4)	7 (5, 7)	0 (0)	22 (22, 23)
Lying	6	4 (4, 5)	2 (2, 2)	2 (2, 3)	0 (0)	9 (8, 10)
Physical aggression	30	16 (10, 20)	3 (3, 3)	5 (4, 7)	0 (0, 0)	24 (20, 30)
Property damage	7	14 (7, 17)	3 (3, 3)	4 (2, 4)	0 (0)	21 (14, 23)
Theft of property	13	6 (5, 10)	2 (2, 2)	2 (2, 4)	0 (0, 0)	12 (9, 16)
Verbal aggression	171	4 (3, 11)	2 (2, 2)	2 (1, 6)	0 (0)	8 (6, 16)
Begging	3	66 (42, 132)	0 (0, 0)	87 (54, 174)	0 (0)	154 (97, 306)
Other	159	2 (2, 3)	2 (2, 2)	1 (0, 1)	0 (0, 0)	5 (4, 6)
Combination	57	9 (3, 43)	2 (2, 2)	5 (1, 29)	0 (0, 0)	20 (6, 110)
**TOTAL**	**508**	**3 (3, 14)**	**2 (2, 2)**	**2 (1, 7)**	**0 (0, 0)**	**7 (5, 26)**

Notes: Med = median; IQR = interquartile range (25th percentile, 75th percentile)

Table S2. Median Monthly Cost per Behavior of Concern on Behavior Plan by Type of Behavior

Behavior Type	Per Month<br>Including Months Where Behavior Was not Observed			Per Month<br>Only Months Where Behavior was Observed		
	Participant Months	Number per Resident per Month<br>Med (IQR)	Societal Cost ($)<br>Med (IQR)	Participant Months	Number per Resident per Month<br>Med (IQR)	Societal Cost ($)<br>Med (IQR)
Health and safety	46	1 (0, 1)	4 (0, 4)	24	2.7 (2.4)	10 (9)
Verbal aggression	387	2 (0, 8)	2 (0, 9)	247	6 (2, 16)	7 (2, 19)
Elopement	66	0 (0, 1)	0 (0, 6)	22	3 (1, 6)	18 (6, 37)
Property damage	126	0 (0, 2)	0 (0, 5)	45	3 (2, 6)	7 (5, 15)
Lying	17	7 (0, 24)	8 (0, 28)	12	17 (5, 33)	19 (5, 38)
Theft of property	63	1 (0, 3)	4 (0, 11)	36	3 (1, 5)	9 (4, 16)
Other	440	2 (0, 13)	2 (0, 15)	258	10 (3, 27)	11 (3, 31)
Physical aggression	255	0 (0, 2)	0 (0, 5)	114	3 (1, 5)	7 (2, 12)
Inappropriate sexual behavior	38	0 (0, 1)	0 (0, 4)	13	7 (1, 13)	25 (4, 47)
Hoarding	24	0 (0, 0)	0 (0)	4	1 (1, 2)	1 (1, 2))
Self-injurious	98	0 (0, 3)	0 (0, 11)	48	3 (1, 8)	11 (4, 29)
**Overall**	**1560**	**8.9 (40.5)**	**12 (48)**	**823**	**5 (2, 14)**	**8 (4, 22)**

Notes: Med = median; IQR = interquartile range (25th percentile, 75th percentile)
